# Patient selection and management for successful cementless total knee arthroplasty

**DOI:** 10.1186/s43019-026-00300-0

**Published:** 2026-01-22

**Authors:** Byung Sun Choi, Min Jung, Byung Woo Cho, Jun Young Chung, Jeong Ku Ha, Hyuk-Soo Han

**Affiliations:** 1 Department of Orthopedic Surgery, Seoul National University Hospital, 28 Yeongeon-dong, Jongno-gu, Seoul, 03080 Republic of Korea; 2 Department of Orthopedic Surgery, Severance Hospital, Yonsei University College of Medicine, Seoul, Republic of Korea; 3 Department of Orthopaedic Surgery, Gangnam Severance Hospital, Yonsei University College of Medicine, Seoul, Republic of Korea; 4 Department of Orthopaedic Surgery, Ajou University Medical School of Medicine, Suwon, Republic of Korea; 5 Seoul Jump Orthopedic Surgery, Seoul, Republic of Korea; 6 Department of Orthopedic Surgery, Seoul National University College of Medicine, Seoul, Republic of Korea

**Keywords:** Arthroplasty, Replacement, Knee, Knee prosthesis, Patient selection, Cementless

## Abstract

Total knee arthroplasty (TKA) is a widely performed procedure for end-stage arthritis, with cemented fixation historically dominating owing to its immediate stability and ability to compensate for minor bone defects. However, concerns over the long-term durability of cemented implants, particularly in younger and more active patients, have renewed interest in cementless TKA as a viable alternative. Advances in implant design, including hydroxyapatite coatings and porous metal surfaces, have improved clinical outcomes, reducing early loosening and enhancing biological fixation. Proper patient selection is crucial for the success of cementless TKA. Studies suggest that younger patients, those with good bone quality, and even some elderly or obese individuals may benefit from cementless implants. While initial migration of the tibial component is more pronounced in cementless TKA, research indicates that this stabilizes over time without impacting long-term outcomes. In addition, pharmacologic interventions, such as bisphosphonates and teriparatide, may help enhance periprosthetic bone density and implant fixation. Despite promising results, challenges remain, particularly in patients with osteoporosis, rheumatoid arthritis, and smoking-related bone health issues. Further research is needed to refine selection criteria, optimize surgical techniques, and ensure long-term success. As next-generation cementless implants continue to evolve, ongoing studies will be essential in guiding patient management strategies for improved outcomes.

In recent decades, total knee arthroplasty (TKA) has been a very effective treatment for patients with end-stage arthritis, showing excellent long-term survival rates, improved pain, and clinical results such as quality of life. According to data from 2010, cemented fixation accounted for approximately 93.5% of TKAs [1]. Cemented fixation provides immediate stability, partially compensates for small bone defects and imperfect flat bone cutting, and can help prevent infection by delivering antibiotics into the joint. However, as the age of patients receiving TKA is gradually decreasing and patients under 65 years of age are predicted to account for more than 50% of TKA patients by 2030, attention to implant survival has been growing [2]. Cement has low resistance to tensile and shear forces and can deform and decompose over the years, causing osteolysis and aseptic loosening. It is known to have a higher failure rate in young or active patient groups [3]. Thus, interest in biological cementless fixation has grown as a promising alternative [4].

Cementless TKA was first introduced in the late 1970s and has attracted interest owing to its advantages of short surgical time, the preservation of bone stock, and the potential for long-term survival owing to biological fixation. However, the clinical results of the early designs of cementless TKA were poor because of early osteolysis, bone resorption, and aseptic loosening [5]. These issues can be alleviated by appropriate patient selection and meticulous bone preparation. Clinical and radiological results have greatly improved since the development of hydroxyapatite (HA) coating and porous metal, and excellent results similar to the results of cemented TKA have been reported (Fig. 1) [6]. Appropriate patient selection and surgical techniques to minimize early loosening and secure longer survival of press-fit implants are essential for the success of cementless TKA (Fig. 2).

**Fig. 1:**
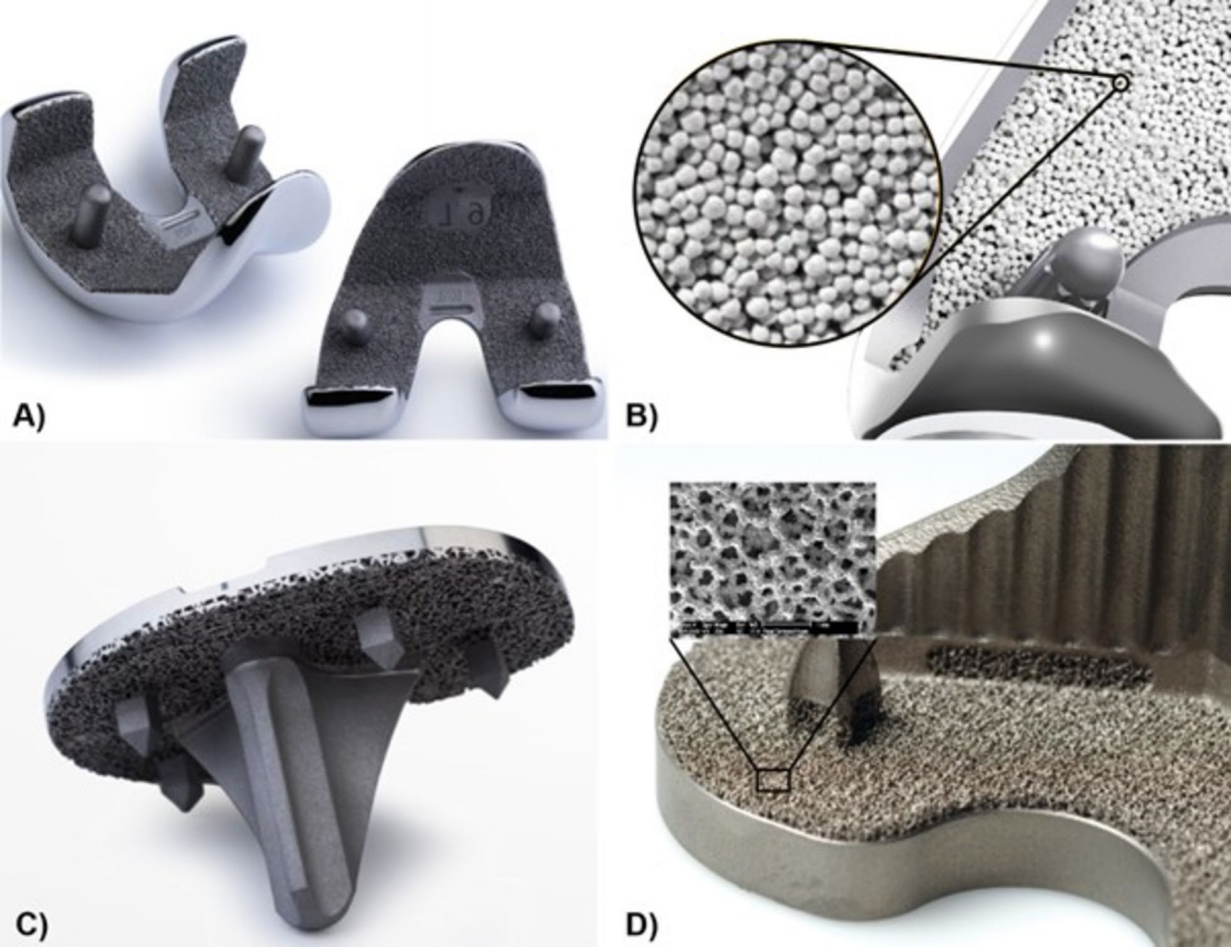
Modern cementless designs promoting biological ingrowth. **A** Plasma spray coating. **B** Hydroxyapatite-coated beads. **C**, **D** Porous coating for biological ingrowth

**Fig. 2:**
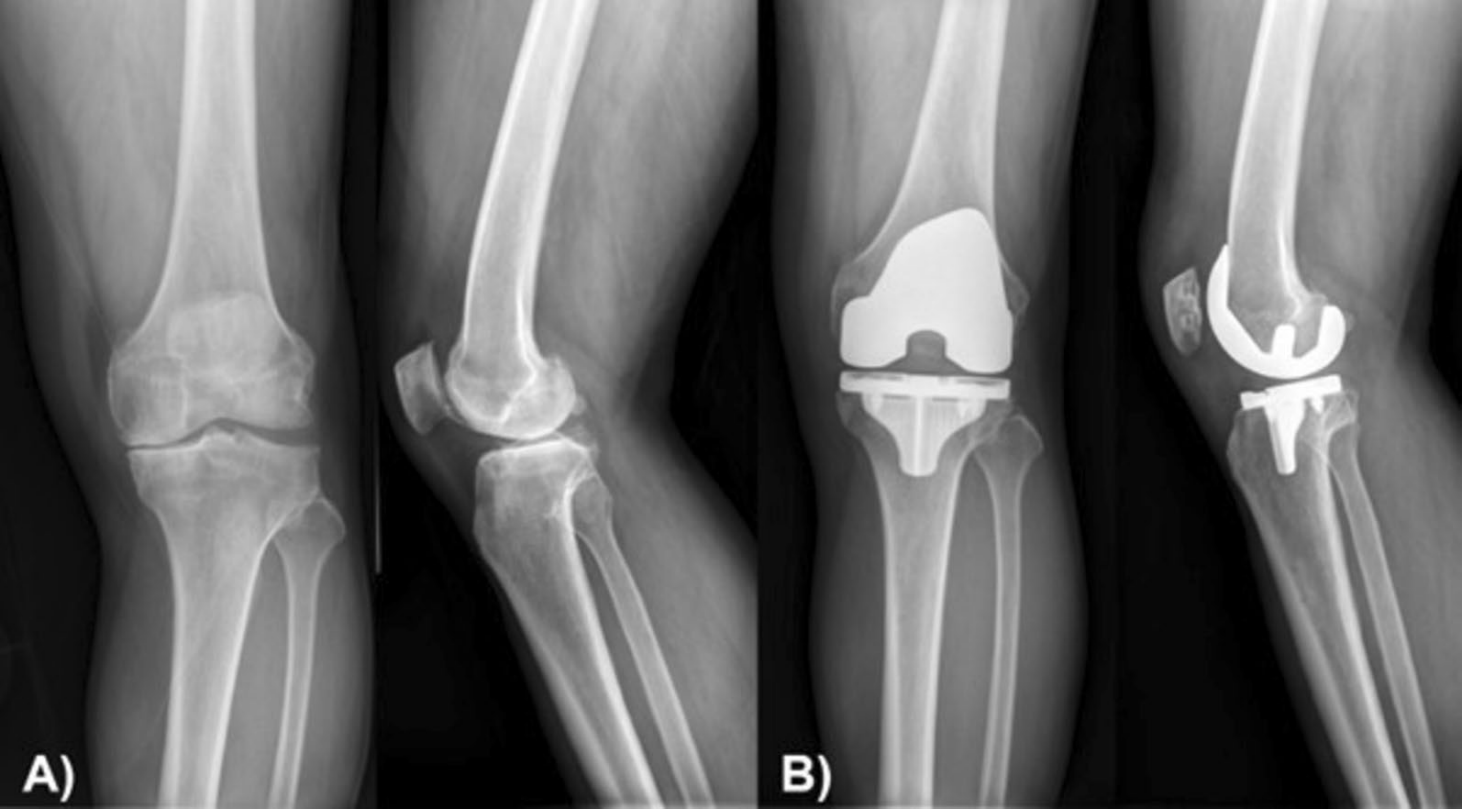
Radiographic images of a 74-year-old male patient with osteopenia and obesity (body mass index [BMI]: 30.33 kg/m^2^) undergoing left cementless total knee arthroplasty. **A** Preoperative X-ray of the left knee. **B** Postoperative X-ray of the left knee

Cementless TKA tends to have slightly more migration of the tibial prosthesis after surgery compared with cemented TKA. A 2-year follow-up randomized controlled trial (RCT) reported by Fricka et al. found no difference in clinical outcomes between the two groups, but the rate of radiolucencies around the prosthesis was higher in the cementless group. In addition, four cases of tibial varus subsidence occurred in the cementless group, showing an average positional change of 3 degrees over 2 years [7]. In a 5-year follow-up study of the same group, no difference in clinical outcomes was found between the two groups, with no additional tibial prosthesis varus subsidence other than those reported at the 2-year follow-up; however, one patient underwent revision surgery owing to aseptic loosening [8]. Recently, many studies using radiostereometric analysis (RSA) techniques have been published, which can accurately measure positional changes in the prosthesis. These studies found that tibial prosthesis migration occurred more frequently in cementless TKA than in cemented surgery and was most frequent in the early stages. In a 5-year follow-up RCT reported by Van Hamersveld et al., the RSA results showed that the cementless group with peri-apatite-coated tibial prostheses had more overall tibial prosthesis migration than the cemented group, which was mainly due to migration during the first 3 months after surgery [9]. In a 2-year follow-up RCT reported by Hasan et al., the RSA results showed that the cementless prosthesis surface treated using a 3D printing technique migrated more than the cemented prosthesis during the first 2 years, and this difference mainly occurred in the first 3 months [10]. In an RCT published by van Ooij et al., three groups (cemented, cementless, and hybrid) with implants with a titanium nitride ceramic coating were compared using RSA [11]. The clinical results were similar among the three groups at 2 years of follow-up, but the cementless group showed more tibial prosthesis movement during the initial 6 months. In a 2-year follow-up RCT, Winther et al. compared two types of cementless tibial prostheses (novel porous titanium surface versus porous plasma spray [PPS]) using RSA. In both groups, migration occurred mainly during the initial 3 months and stabilized thereafter [12]. Less subsidence occurred from 12 to 24 months in the novel porous titanium surface group, and less migration occurred during the initial 3 months in the PPS group. No difference in migration between the two groups was seen at 2 years. A 5-year follow-up study comparing the same patient group showed no further differences [13]. Several other RSA research studies have reported similar results. Most of them reported observing a small amount of displacement in the first 3 months and stabilization after about 1 year (Fig. 3) [14, 15]. However, since a small amount of displacement is observed in cemented TKA and some studies have reported no difference in displacement between cemented and cementless, it seems difficult to say that it is a disadvantage only in cementless TKA [16, 17].

**Fig. 3:**
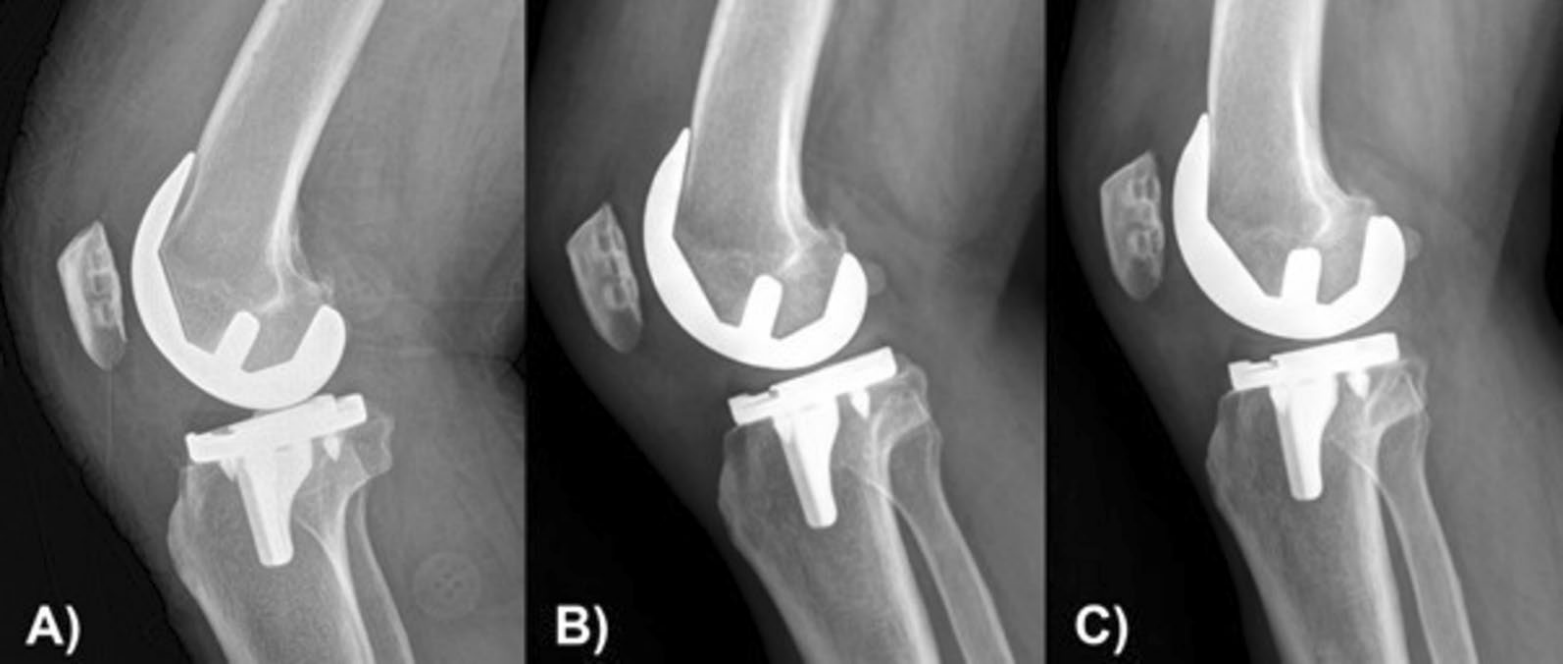
Radiographic images of a 74-year-old male patient with osteopenia and obesity (BMI: 30.33 kg/m^2^) undergoing left cementless total knee arthroplasty. **A** Immediate postoperative X-ray showing initial implant positioning with visible gaps between the bone and implant. **B** Postoperative X-ray at 3 months, demonstrating gap healing and the beginning of osseointegration as the bone–implant interface starts to stabilize. **C** Postoperative X-ray at 1 year, with complete osseointegration and optimal implant fixation, indicating successful biological fixation and stable bone–implant interaction

## Age

**Fig. 4:**
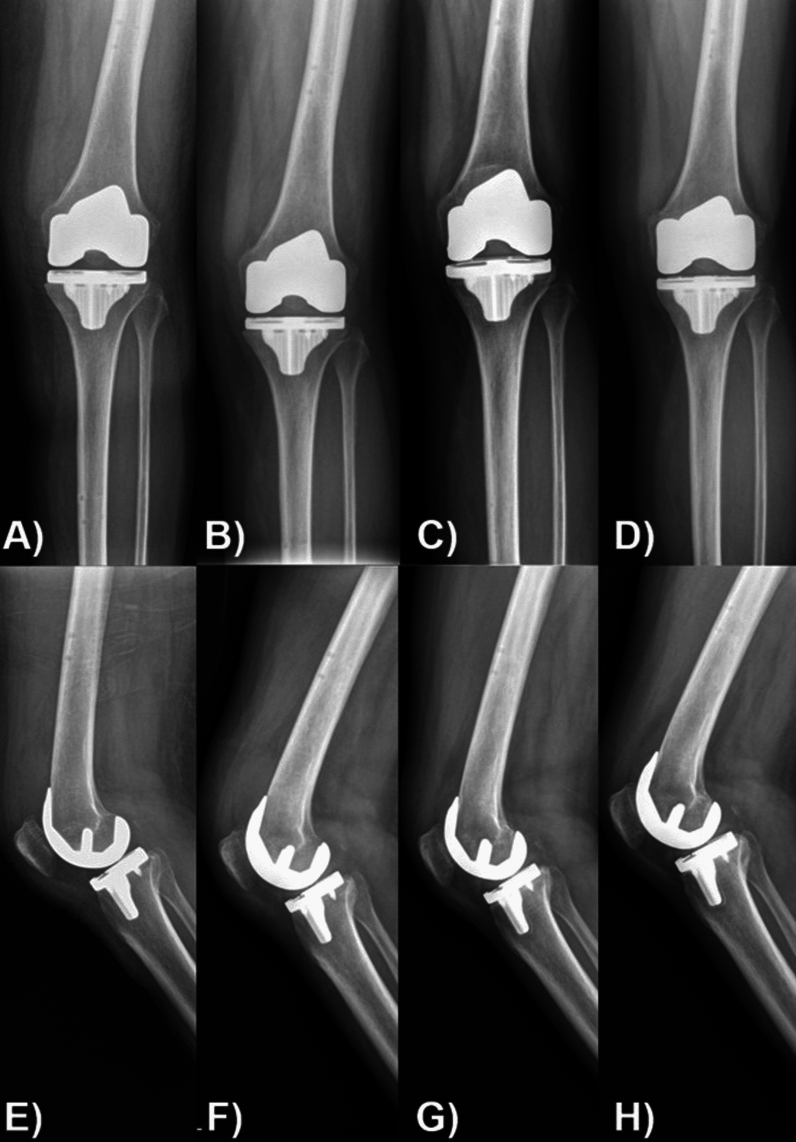
Radiographic images of a 64-year-old female patient with osteopenia and overweight (BMI: 27.04 kg/m^2^) undergoing left cementless total knee arthroplasty demonstrate progressive posterolateral subsidence of the tibial baseplate over time, particularly when comparing the serial anteroposterior (**A**–**D**) and lateral (**E**–**H**) views. **A**, **E** Immediate postoperative X-rays; **B**, **F** 1 month postoperative; **C**, **G** 6 months postoperative; and **D**, **H** 1 year postoperative

Concerns about early migration or loosening (Fig. 4) are particularly relevant in older patients. In osteopenic bones in elderly patients, primary stability and osseointegration at the bone–implant interface may be questionable, and the bone may not be able to withstand the load required by a cementless implant [18]. This especially applies to elderly women, as highlighted by the American Joint Replacement Registry (AJRR) finding that women aged 65 years and older had an increased rate of revision after cementless TKA [19]. However, many studies have demonstrated the opposite results. A single-center retrospective review by Newman et al. evaluated 142 TKAs in both men and women aged 75 years and older and found a 4-year survival rate of over 99% with no major radiolucencies, loosening, or prosthesis subsidence [20]. Another retrospective cohort study evaluated women aged 75 years and older who underwent mobile-bearing, cementless TKA, where subsidence was observed in four of 1000 TKAs, and none required operative management [21]. A retrospective matched cohort study comparing 120 cementless TKAs with 360 cemented TKAs of the same design in patients aged 75 years or older found no difference in functional scores after 2 years [22]. At 7 year follow-up, survival was 99.4% for cemented knees and 100% for cementless knees. In a retrospective study, Gomez et al. reported that cementless TKA is a valid option for patients aged 70 years or older, with favorable clinical, radiological, and survival outcomes [23]. Similarly, a retrospective cohort of 347 cementless TKAs of different age groups reported no revisions owing to aseptic loosening. Patients aged 70 years or older could achieve clinical outcomes 2 years after cementless TKA comparable to those in younger patients [24]. Newer-generation implants are expected to improve osseointegration at the biological interface between bone and prosthetic components, even in elderly patients; however, longer-term survival studies are needed.

A case series of new-generation cementless TKAs in patients younger than 50 years of age reported a 100% implant survivorship at a mean follow-up of 4 years [25]. A systematic review and meta-analysis of six RCTs on young patients under 65 years old compared cemented and cementless TKAs over a mean 12-year follow-up. The clinical outcomes of cemented TKA in young patients were superior to cementless TKA, although no significant difference was found in complications, aseptic loosening, or reoperation rates [26]. Most recently, cementless tibial replacements with surfaces treated with PPS or 3D printing techniques have been released and are also showing excellent results. In a 2-year follow-up RCT reported by Nam et al., when a highly porous titanium-coated cementless tibial replacement using 3D printing was compared with a cemented tibial replacement, no difference in clinical or radiological outcomes was observed between the two groups, and no subsidence or aseptic loosening of the replacements occurred [27].

## Obesity

Concerns about the use of cementless TKA in obese patients have been raised. Traditional cementing techniques have been shown to increase reimplantation rates in obese patients owing to loosening and subsidence [28]. In a large retrospective observational cohort study, Abdel et al. calculated the risk of revision due to aseptic tibial loosening associated with increased bone mass index (BMI) in 5088 TKAs. They showed that a BMI of more than 35 kg/m^2^ was associated with a twofold higher risk of tibial failure. Similarly, a retrospective study by Vazquez-Vella Johnson et al. showed that obesity and age younger than 60 years were associated with increased failure rates [29]. The mechanism of failure was related to increased shear and stress at the bone–cement interface, leading to micromotion and aseptic loosening or osteolysis [30]. However, a multi-cohort study of patients with BMIs  ≥ 30, ≥ 35, and ≥ 40 found no significant difference in outcomes or reimplantation rates between cemented and cementless fixation, with survival rates in both groups being excellent and possibly improved in the cementless group [31–33]. In a retrospective comparative study, Bagsby et al. investigated 292 TKAs (154 cemented and 245 cementless) in patients with BMIs of more than 40 kg/m^2^ [30]. They found a higher incidence of aseptic loosening in the cemented group (5.8%) compared with the cementless group (0%), and suggested that, given the higher mechanical stresses, biological growth with cementless implants may be more durable and better tolerated in obese patients. Another retrospective study comparing 108 cementless and 85 cemented TKAs in morbidly obese patients (BMI > 40 kg/m^2^) found a higher 8-year survival rate with cementless fixation (99.1% versus 88.2%) [32]. The authors concluded that cementless fixation is a promising alternative for obese patients.

## Osteoporosis

Because cementless TKA relies on primary stability and osseointegration, poor bone quality, such as osteoporosis or osteopenia, can be problematic [18]. General and periarticular bone health appears to be relevant to the long-term outcomes of patients undergoing cementless TKA, with implant stability possibly being reduced in patients with poor bone quality [16]. This finding was confirmed in both laboratory and clinical settings. A laboratory study found greater tibial micromotion in osteoporotic bone models [34]. A previous prospective cohort study by Petersen et al. found greater peak tibial component motion in patients with lower tibial bone mineral density (BMD) after 2 years, and a more recent prospective study using RSA and dual-energy X-ray absorptiometry (DEXA) found similar results [35, 36]. They reported that the greatest displacement (up to 1 mm) occurred within 1 year after surgery and also revealed that bone density and implant displacement were positively correlated, concluding that the higher the bone density, the less displacement occurred. However, a prospective cohort study of 100 patients found no difference in tibial component migration between cemented and cementless knee arthroplasty with respect to bone density or bone turnover markers, although lower preoperative vitamin D levels were associated with greater tibial component migration in all patients [37]. A retrospective study on cementless TKA in patients with osteonecrosis had a survival rate of greater than 97%, and no loosening was detected for at least 3 years after surgery [38]. The study concluded that excellent implant survival and clinical and radiological outcomes can be expected after primary cementless TKA for knee osteonecrosis.

## Rheumatoid arthritis and smoking

Patients with rheumatoid arthritis (RA) and those who are persistent smokers are also theoretically at increased risk after cementless TKA owing to poor bone quality and the possible inhibition of bone growth [39]. The inherent inflammatory process in the background of RA and the chronic use of medications such as corticosteroids have been suggested as contributing factors [40]. Conversely, recent advances in biological and immunomodulatory therapies have changed the disease status of patients with RA, with several studies reporting positive results in improving BMD [41]. However, in a retrospective cohort study by Patel et al. found a survival rate of greater than 99% in 122 patients with RA (126 cementless TKAs) at a mean follow-up of 4 years [42]. They suggested that the advancements of cementless component fixation do not constitute a definite contraindication for patients with RA. Another retrospective study following 112 patients with cementless TKA for RA also found a survival rate of greater than 95% at 15 years of follow-up, suggesting that the cementless technique is a viable option for patients with RA with good clinical outcomes and no serious complications [43]. Similarly, the vasoconstrictive effect of nicotine and the reduced oxygen-carrying capacity associated with increased carboxyhemoglobin concentrations may have a negative effect on biological bone function. Nicotine has been shown to reduce osseointegration to porous titanium and HA implants in animal studies [44, 45]. However, a retrospective review of 293 consecutive cementless TKAs found that active tobacco use may have interfered with the biological fixation of the component but did not increase the rate of revision or aseptic loosening at 2.5 years postoperatively [46].

## Periarticular bone evaluation

A recent retrospective query of national administrative claims databases found that non-elderly patients with osteoporosis had similar medical and implant-related complications and implant survival rates after cementless and cemented TKA [47]. However, a retrospective database study by Dubin et al. reported an increased risk of periprosthetic fractures at 5 years following TKA in patients at high risk for osteoporosis undergoing cementless fixation compared with cemented fixation [48]. Therefore, intraoperative decisions regarding bone quality are needed to reliably distinguish between candidates for cementless TKA and those who are not. In a retrospective study, Choi et al. suggested that volumetric BMD assessment using dual-energy computed tomography was an accurate, relatively simple, and useful tool for osteoporosis screening before cementless TKA in clinical practice [49].

## Medications on osteoporotic bone

The guidelines for pharmacologic therapy in patients undergoing elective TKA are not clearly defined. Also, the preservation of periprosthetic bone mass to improve survival in TKA has been an important topic, especially in the context of cementless fixation. Periprosthetic BMD after TKA varies with alignment correction and changes in mechanical loading of the knee after TKA implantation, and several studies have reported decreased periprosthetic BMD after TKA [12]. A meta-analysis by Lin et al. found that patients who started bisphosphonates at or shortly after TKA had significantly higher BMD at the proximal tibial shaft and distal femur at 3, 6, and 12 months postoperatively compared with controls [50]. The clinical implications of medication have also been explored with respect to long-term outcomes. Several studies have shown that increased BMD may contribute to improved implant fixation and a reciprocal decrease in revision rates up to 5 years in patients in whom antiresorptive therapy is initiated after TKA [51]. This national data study reviewed 331,660 TKA patients with 4–14 years of follow-up and found an overall population revision rate of 1.4% in bisphosphonate users compared with 2.9% in non-bisphosphonate users. In a large database study, Namba et al. found that the overall rate of aseptic revision was lower in the bisphosphonate group (0.5%) than in the non-bisphosphonate group (1.6%) at a mean follow-up of 3.7 years [52]. This effect was independent of bone quality categories in those older than 65 years. Of note, these studies included significantly more cemented than cementless TKA patients, and none of the studies stratified the two groups in their reports. Little data is available on the effects of antiresorptive medications in cementless TKA.

A case–control study included 8 men and 32 women (mean age, 75.6 years) who underwent cementless TKA for medial knee osteoarthritis with (*n* = 20) or without (*n* = 20) once-weekly subcutaneous/hypodermic injections of teriparatide for 48 weeks [53]. They evaluated BMD and bone volume at the bone-prosthesis interface of the proximal tibia using multi-detector computed tomography. Weekly injections of teriparatide after cementless TKA promoted bone ingrowth, mostly in the medial aspect of the bone-prosthesis interface.

## Patient selection framework

For clinical applicability, Table 1 summarizes key selection parameters for cementless TKA, integrating demographic, mechanical, and biological factors. This decision-making framework is designed to assist surgeons in identifying optimal candidates and adjusting perioperative strategies accordingly.

**Table 1:** Summary of patient selection framework for cementless total knee arthroplasty

Patient category	Characteristics	Considerations
Ideal candidates	- Younger (< 65 years) - Active patient - Good bone quality (normal BMD)	- Strong evidence supports cementless TKA for potential long-term biological fixation and durability, avoiding late cement failure
Suitable candidates (with considerations)	- Elderly (65 ≤ age < 75 years) - Obese (BMI > 30 kg/m^2^) - Well controlled RA	- Elderly: multiple retrospective studies show excellent outcomes in select patients with good bone stock. Intraoperative assessment of bone quality is crucial - Obesity: retrospective studies suggest cementless fixation may offer superior durability compared with cemented fixation, potentially reducing the risk of aseptic loosening under high mechanical stress - RA: modern cementless designs show high survival rates in patients with well-managed RA, though theoretical risks related bone quality persist
Relative contraindications (use with caution)	- Advanced age (≥ 75 years) - Osteoporosis - Active smokers - Bone defects or poor bone quality	- Age: in patients with limited life expectancy, the biological advantages of cementless fixation may not provide meaningful benefit - Osteoporosis: lower BMD is correlated with increased initial micromotion. Preoperative BMD assessment is recommended - Smoking: animal studies show nicotine impairs osseointegration - Bone quality: the ultimate decision may rely on the surgeon’s intraoperative assessment. If bone quality is deemed insufficient to achieve stable primary fixation, converting to a cemented implant is a safe and reliable option

*TKA* total knee arthroplasty, *BMD* bone mineral density, *BMI* body mass index, *RA* rheumatoid arthritis

For clinical implementation, we propose a concise perioperative protocol for cementless TKA. Preoperatively, a thorough assessment should include risk factors such as age, activity level, BMI, RA control, and smoking status. Bone quality, alignment, and deformity can be evaluated using DEXA, and any vitamin D deficiency should be corrected. Shared decision-making is key in discussing the final fixation choice. Intraoperatively, the surgeon must confirm primary stability through press-fit engagement and stable varus–valgus testing, and avoid component underhang or overhang. If metaphyseal bone is poor or stability is inadequate, conversion to cemented or hybrid fixation is prudent. Postoperatively, patients can bear weight as tolerated, with serial radiographs to monitor the implant. While small, early radiolucencies or migrations typically stabilize within a year, progressive changes warrant closer surveillance. Medication for osteoporosis should be considered for patients with severe osteoporosis.

## Conclusions

Recently introduced next-generation cementless TKA is showing promising clinical results by addressing the causes of previous failures through the integration of advanced technologies. For successful application, careful patient selection and management are crucial. While current evidence is encouraging, a significant knowledge gap remains regarding the long-term survivorship of these modern implants, particularly in high-risk groups such as obese or osteoporotic patients. Future studies are essential to address this issue and build definitive, evidence-based confidence in cementless TKA for a broader range of patients.

## Data Availability

Review articles typically do not generate new datasets. The datasets used and/or analyzed during the current study are available from the corresponding author on reasonable request.
